# Trends in inpatient antiparkinson drug use in the USA, 2001–2012

**DOI:** 10.1007/s00228-015-1881-4

**Published:** 2015-06-18

**Authors:** James A. G. Crispo, Yannick Fortin, Dylan P. Thibault, Matthew Emons, Lise M. Bjerre, Dafna E. Kohen, Santiago Perez-Lloret, Donald Mattison, Allison W. Willis, Daniel Krewski

**Affiliations:** McLaughlin Centre for Population Health Risk Assessment, University of Ottawa, Ottawa, ON Canada; Fulbright Canada Student, University of Pennsylvania, Philadelphia, PA USA; Departments of Neurology and Biostatistics & Epidemiology, University of Pennsylvania Perelman School of Medicine, Philadelphia, PA USA; Cerner Corporation, Culver, CA USA; C.T. Lamont Primary Health Care Research Centre, Department of Family Medicine, University of Ottawa, Ottawa, ON Canada; Bruyère Research Institute, Ottawa, ON Canada; School of Epidemiology, Public Health and Preventive Medicine, University of Ottawa, Ottawa, ON Canada; Clinical Pharmacology and Epidemiology, Catholic University, Buenos Aires, Argentina; Risk Sciences International, Ottawa, ON Canada

**Keywords:** Parkinson’s disease, Cohort study, Drug utilization, Medical records, Pharmacoepidemiology

## Abstract

**Purpose:**

Although therapeutic options and clinical guidelines for Parkinson’s disease (PD) have changed significantly in the past 15 years, prescribing trends in the USA remain unknown. The purpose of this population-based cohort study was to examine patterns of inpatient antiparkinson drug use between January 2001 and December 2012 in relation to clinical guideline publication, drug introduction/withdrawal, and emerging safety concerns.

**Methods:**

A total of 16,785 inpatients receiving pharmacological treatment for PD were identified in the Cerner Health Facts database. Our primary outcome was standardized (age, sex, race, and census region) annual prevalence of antiparkinson drug use. We also examined antiparkinson medication trends and polypharmacy by age and sex.

**Results:**

The most frequently prescribed antiparkinson drugs between 2001 and 2012 were levodopa (85 %) and dopamine agonists (28 %). Dopamine agonist use began declining in 2007, from 34 to 27 % in 2012. The decline followed publication of the American Academy of Neurology’s practice parameter refuting levodopa toxicity, pergolide withdrawal, and pramipexole label revisions. Despite safety concerns for cognitive impairment and falls, individuals ≥80 years of age demonstrated stable rates of dopamine agonist use from 2001 to 2012. Polypharmacy was most common in younger patients.

**Conclusions:**

Dopamine agonist use declined from 2007 to 2012, suggesting that increased awareness of safety issues and practice guidelines influenced prescribing. These events appear to have minimally influenced treatment provided to older PD patients. Antiparkinson prescribing trends indicate that safety and best practice information may be communicated effectively.

**Electronic supplementary material:**

The online version of this article (doi:10.1007/s00228-015-1881-4) contains supplementary material, which is available to authorized users.

## Introduction

Treatment options for both early and advanced Parkinson’s disease (PD) have expanded considerably over the last 15 years. The introduction of dopamine agonists (DAs) to treat PD in the late 1990s, USA Food and Drug Administration approval of deep brain stimulation for PD in 2002, and approval of other antiparkinson drugs such as entacapone and rasagiline reflect the substantial public and private investment in improving the lives of those affected by PD. Increasing knowledge of antiparkinson drug safety (including reports of DA-associated impulse control disorders [1, 2] and cardiotoxicity [3–9]) and efficacy, findings that PD progression is not accelerated by levodopa [10, 11], and the failures of selegiline and rasagiline to demonstrate neuroprotection [11, 12] have the potential to influence clinical practice.

Ideally, prescribing practices reflect known risks and benefits, which can change over time. Evidence-based clinical guidelines are routinely published and updated by professional organizations such as the International Parkinson and Movement Disorder Society and the American Academy of Neurology (AAN) to reflect changes in scientific knowledge [11, 13–15]. Additionally, treatment availability and adverse drug event reporting may also contribute to changing antiparkinson drug prescribing practices over time. Previous USA PD drug utilization studies have consistently demonstrated sociodemographic disparities in PD treatment [16–18]; however, prescribing trends are unreported. Furthermore, the impact of evidence-based guideline publication, drug availability, and safety concerns on antiparkinson drug prescribing has been investigated in Europe, Asia, and Australia [19–22], but not in the USA. Previous studies have shown that levodopa is the most commonly prescribed antiparkinson drug [19–22], that prescribing of DAs may be influenced by safety concerns [20], and that despite safety concerns, DAs may be routinely prescribed to older adults [22]. Nevertheless, changes in antiparkinson drug use in relation to practice guideline publication, drug availability, and emerging safety concerns remain unknown in the USA.

To address this knowledge gap, we performed a 12-year retrospective analysis of electronic medical records (EMRs) from more than 16,000 individuals with PD in Cerner Health Facts^®^, an EMR database comprised of complete encounter data for patients who received care at any USA health center subscribed to Cerner EMR services. The primary objective of our study was to describe patterns of antiparkinson drug use between January 2001 and December 2012 in relation to clinical guideline publication, drug introduction/withdrawal, and emerging safety concerns. PD prevalence increases sharply with age [23], and yet older adults are also most vulnerable to side effects from PD medications [2, 6, 24]. Our secondary objectives were therefore to examine temporal trends and relative differences in the pharmacological management of PD by age and sex.

## Materials and methods

This study was approved by the Health Sciences and Science Research Ethics Board at the University of Ottawa, Ottawa, ON, Canada.

### Data source

Study data were derived from the Cerner Corporation’s (Kansas City, Missouri) Health Facts^®^ data warehouse. Launched in January 2000, Health Facts^®^ is an electronic medical record system that stores time-stamped patient records, including sociodemographic, geographical, clinical, laboratory, pharmacy, and billing data for clients. As of 2014, there were over 300 contributing subscribers to the Health Facts^®^, and data from more than 230 million patient encounters, representing over 41 million distinct patients. Health Facts^®^ subscribers are situated in all USA census regions: Northeast (40 %), Midwest (27 %), South (21 %), and West (11 %). The majority of subscribers are academic medical centers, which contribute approximately 65 % of all encounters. Health Facts^®^ is well suited for studying responses to changing practice guidelines, specifically among inpatients, since pharmacy data are most complete for inpatient populations.

### Study population

We searched all encounters from January 1, 2000, to December 31, 2012, to identify individuals with a primary or secondary diagnosis of PD according to the International Classification of Diseases, Ninth Revision (ICD-9; code 332 for PD, or code 332.0 for Paralysis Agitans). Information from all encounters within the study period was extracted for individuals with one or more PD diagnoses (*n* = 40,609). We excluded individuals diagnosed at any time with a primary or secondary diagnosis of secondary parkinsonism (ICD-9, code 332.1) or other degenerative diseases of the basal ganglia (ICD-9, code 333.0) (*n* = 1,450, 3.6 %). In effort to exclude individuals with atypical PD and cases of PD misclassification, we also excluded those who were diagnosed with PD prior to age 40 (*n* = 375, 0.96 %) and those without a recorded age at time of PD diagnosis (*n* = 18, 0.05 %). Because all outpatient physicians do not use Health Facts^®^, we further restricted the cohort to inpatients who were prescribed one or more antiparkinson drugs between January 1, 2001, and December 31, 2012 (*n* = 17,375, 45 %). Finally, to accommodate direct standardization, we restricted the cohort to individuals with complete demographic information (age, sex, and race) recorded (*n* = 16,785, 97 %).

### Demographic data and care setting characteristics

Demographic data collected from encounters included patient age, sex (male or female), and race (Caucasian, African American, Asian, Hispanic, or other). Patient age at time of first recorded PD diagnosis, defined as the study-qualifying encounter, was categorized into the following age strata: 40–64, 65–79, and 80+ years. The principal diagnosis for each study encounter was identified and classified according to commonly used ICD-9 categories. Care setting characteristics collected from study-qualifying encounters included location type (urban or rural), teaching status (teaching or nonteaching), and census region (Northeast, South, Midwest, or West).

### Drug utilization

Our primary outcome was standardized (age, sex, race, and census region) annual prevalence of antiparkinson drug use among inpatients with PD who were prescribed one or more antiparkinson drugs. Antiparkinson drugs were identified by searching hospital formularies for generic names of interest and classified according to the following categories: (1) levodopa, (2) DA (ergot (bromocriptine, cabergoline, and pergolide) and nonergot (pramipexole, ropinirole, and rotigotine)), (3) monoamine oxidase-B (MAO-B) inhibitor (selegiline and rasagiline), (4) catechol-*o*-methyltransferase (COMT) inhibitor (tolcapone and entacapone), (5) amantadine, and (6) anticholinergic (benztropine, biperiden, procyclidine, and trihexyphenidyl). After excluding drug orders that were cancelled or not dispensed, the number of inpatients with an antiparkinson prescription was calculated annually by drug class from January 1, 2001, to December 31, 2012. Using data from the 2005 American Community Survey (USA Census Bureau), we calculated annual standardized (age, sex, race, and census region) antiparkinson drug use to examine prescribing trends over time. Subgroup analyses contrasted prescribing trends by age and sex. We defined PD drug complexity as the sum of unique drug classes prescribed to an individual patient in one calendar year, categorized as 1, 2, 3, and 4+ drugs.

### Factors affecting prescribing practice

Our a priori hypothesis was that the greatest changes in the most commonly prescribed drug classes—levodopa and DAs—would be temporally related to (1) practice guideline publication, (2) official safety concerns (such as market withdrawal), and (3) the 2008 pramipexole (Mirapex) label revisions warning of the risk of impulse control issues (urges to gamble and increased sexual urges). To test this hypothesis, we examined the change in levodopa and DA use between the event year and 1, 2, and 3 years after (1) publication of the April 2006 AAN practice parameter reporting that levodopa does not accelerate PD progression and that no pharmacological intervention is neuroprotective, (2) the voluntary withdrawal of pergolide (an ergot-derived DA) from the market in 2007 due to concerns about cardiotoxicity, and (3) the December 2008 pramipexole label revisions which added precautions about uncontrollable urges to the label. We used a Wilcoxon-Mann-Whitney test to examine whether standardized annual prevalence of levodopa and DA use significantly changed after events of interest. In order to ensure independence of the data between the two years being compared, individuals appearing in both years (6–25 % of sample depending on the particular comparison) were excluded from the analyses. Two-tailed *p* values less than 0.0015 were considered statistically significant.

## Results

### Demographics and care setting characteristics

We identified 16,785 individuals with PD from the Cerner Health Facts^®^ data warehouse who satisfied our inclusion/exclusion criteria between January 1, 2001, and December 31, 2012 (Table 1). The demographic characteristics of our population were similar to previously published epidemiological studies of PD in the USA [23, 25]. Caucasians comprised 91.2 % of the population; the remaining individuals were African-American (6.1 %), Asian (0.7 %), Hispanic (1.0 %), and other races (1.0 %). Men (54.9 %) were more prevalent than women (45.1 %) (Table 1). The majority (88.4 %) of individuals were aged 65 years or older at the time of their first recorded PD diagnosis in Health Facts^®^, which agrees with published data on age-stratified PD prevalence [23, 25]. Care centers were most likely located in urban areas (99.6 %), academic medical centers (64.9 %), and in the Northeast USA (49.2 %). Supplementary Table 1 shows that study cohort demographics and care setting census regions were similar across study years. Supplementary Table 2 demonstrates that individuals within our study cohort were primarily admitted to hospital for diseases of the circulatory system, diseases of the respiratory systems, and symptoms, signs, and ill-defined conditions, which is consistent with reasons for inpatient admission among older USA adults [26].

**Table 1:** Demographics of inpatients with PD and care setting characteristics

	Population	
Characteristic	*n* (16,785)	%
Age at diagnosis^a^		
40–64	1943	11.6
65–79	7574	45.1
80+	7268	43.3
Sex		
Male	9211	54.9
Female	7574	45.1
Race		
Caucasian	15,314	91.2
African-American	1026	6.1
Asian	114	0.7
Hispanic	167	1.0
Other	164	1.0
Care setting		
Urban	16,726	99.6
Rural	57	0.3
Teaching status		
Teaching	10,899	64.9
Nonteaching	5886	35.1
Census region		
Northeast	8254	49.2
South	4225	25.2
Midwest	3007	17.9
West	1299	7.7

*PD* Parkinson’s disease

^a^Age at first recorded diagnosis in Health Facts

### Changes in drug utilization in relation to AAN practice guidelines

**Fig. 1:**
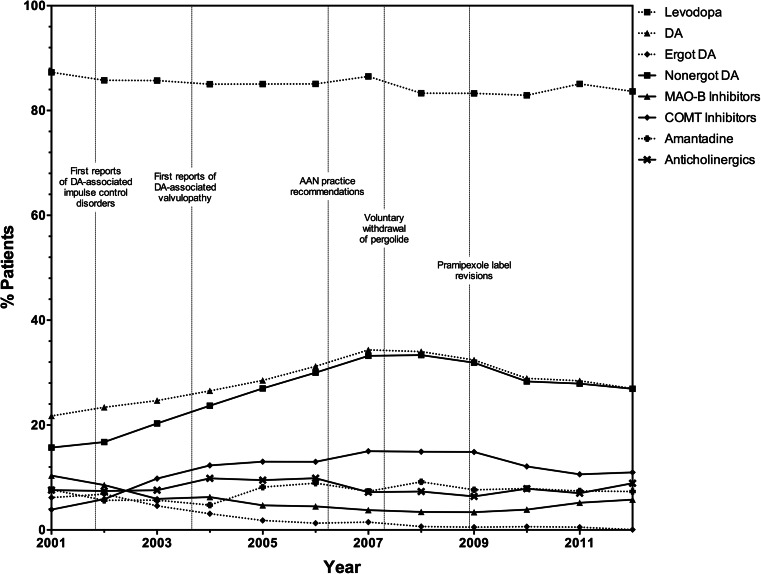
Standardized prevalence of antiparkinson drug use over time. *AAN* American Academy of Neurology, *COMT* catechol-*o*-methyltransferase, *DA* dopamine agonist, *MAO-B* monoamine oxidase-B

As shown in Fig. 1 and Table 2, use of levodopa was stable prior to and after the 2006 publication of the AAN’s evidence-based review of neuroprotective strategies and alternative therapies in PD, which put forth that there was no advantage to initiating therapy with levodopa alternatives. DAs were the most commonly used levodopa alternatives at that time, and DA utilization steadily increased from 21.7 % (2001) to 31.2 % (2006) during the same pre-guideline period. Use of nonergot DAs was significantly higher (+3.2 %; *p* < 0.0015) in the year immediately following release of the AAN practice guideline; however, did not further increase in subsequent years (Table 2). Prevalent use of ergot DAs agonists significantly declined 2 (−0.6 %; *p* < 0.0015) and 3 years (−0.8 %; *p* < 0.0015) after AAN practice guideline publication (Table 2).

**Table 2:** Change in levodopa and dopamine agonist utilization in relation to guideline publication, pergolide withdrawal, and emerging safety concerns

Event	Event year (%^a^)	Year after (%^a^)	% Change*	2 years after (%^a^)	% Change*	3 years after (%^a^)	% Change*
AAN practice recommendations (2006)							
Levodopa	85.1	86.5	+1.4	83.3	−1.8	83.3	−1.8
Dopamine agonists	31.2	34.4	+3.2	34.0	+2.8	32.4	+1.2
Ergot	1.3	1.5	+0.2	0.7	−0.6*	0.5	−0.8*
Nonergot	30.0	33.2	+3.2*	33.4	+3.4	31.9	+1.9
Pergolide withdrawal (2007)							
Levodopa	86.5	83.3	−3.2	83.3	−3.2	82.9	−3.6
Dopamine agonists	34.4	34.0	−0.4	32.4	−2.0	28.9	−5.5*
Ergot	1.5	0.7	−0.8	0.5	−1.0	0.6	−0.9
Nonergot	33.2	33.4	+0.2	31.9	−1.3	28.3	−4.9*
Pramipexole label revisions (2008)							
Levodopa	83.3	83.3	0.0	82.9	−0.4	85.1	+1.8
Dopamine agonists	34.0	32.4	−1.6	28.9	−5.1	28.5	−5.5*
Ergot	0.7	0.5	−0.2	0.6	−0.1	0.5	−0.2
Nonergot	33.4	31.9	−1.5	28.3	−5.1	27.9	−5.5*

Due to polypharmacy, the sum of the standardized annual prevalent use for antiparkinson drugs studied may exceed 100 % in a given year

*AAN* American Academy of Neurology

^a^Standardized annual prevalent use expressed as a percentage

*Significant at *p* = 0.0015 ≈ 0.05/36, using a Bonferroni adjustment to control for the 36 individual tests conducted

### Changes in drug utilization in relation to pergolide withdrawal

In March 2007, pergolide (an ergot DA) was withdrawn from the USA market over mounting evidence of cardiotoxicity, specifically valvular disease [27]. Use of DAs (mainly nonergot DAs) surged prior to the withdrawal of pergolide, increasing from 21.7 % in 2001 to a maximum of 34.3 % in 2007 (Fig. 1). At the time of withdrawal, approximately 1.5 % of inpatients receiving pharmacotherapy for PD were treated with ergot DAs. Levodopa use was not impacted by the withdrawal of pergolide (Table 2). Similarly, the withdrawal of one of its class members had little immediate impact on DA use; however, DA (−5.5 %; *p* < 0.0015) and nonergot DA (−4.9 %; *p* < 0.0015) use significantly declined 3 years following pergolide’s withdrawal (Table 2).

### Changes in drug utilization in relation to pramipexole label revisions

In-depth precautions about uncontrollable urges were added to the pramipexole (Mirapex) label in 2008 [28]. Moreover, 2008 was a focal year in the use trend of DAs, as it was the first year in our sample where DA use decreased (−1.6 %; *p* > 0.0015) from the previous year (Fig. 1 and Table 2). A significant decrease (−5.5 %; *p* < 0.0015) in the use of nonergot DAs was observed 3 years following the pramipexole label revisions, while use of levodopa remained unchanged (Table 2).

### Prescribing trends by age and sex

**Fig. 2:**
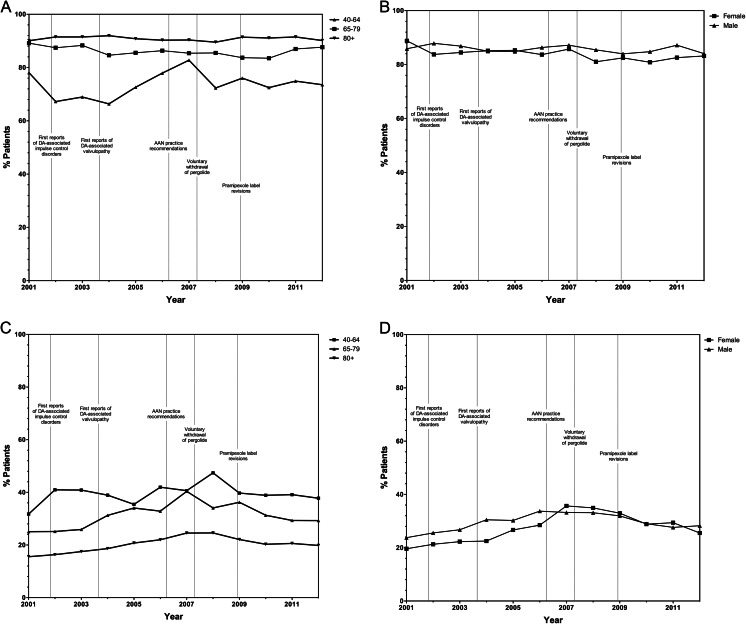
Standardized prevalence of antiparkinson drug use over time by age and sex. Levodopa use over time by age (**a**) and sex (**b**), and dopamine agonist use over time by age (**c**) and sex (**d**). *AAN* American Academy of Neurology, *DA* dopamine agonist. Trends were not standardized by the stratification variable for analyses of annual prevalence of antiparkinson drug use by age and sex

No discernable differences according to age or sex were observed in the use trends of levodopa or DAs following (1) publication of the 2006 AAN practice parameter, (2) the 2007 withdrawal of pergolide from the USA market, and (3) the 2008 pramipexole labeling revisions (Fig. 2). We observed differences in levodopa (Fig. 2a) and DA (Fig. 2c) use within older adult PD populations (80+ years of age). On average, 73.4 % of adults aged 40–64 years used levodopa, which rose to 90.1 % for individuals aged 80+ years. Younger patients (40–64 years of age) were uncommon and were more likely to be prescribed DAs, which decreased with increasing age (Fig. 2c). Adults aged 65–79 years had intermediate rates of DA and levodopa use, with levodopa favored over DAs. As shown in Fig. 2, year-to-year trends in levodopa and DA use were more volatile for younger patients, while the pharmacological management of PD proved to be more resistant to change among older patients. Despite emerging safety concerns pertaining to the use of DAs in older populations, use of DAs did not decrease over time in the oldest (80+ years of age) population, 20.2 % of who were prescribed a DA. Polypharmacy (two or more PD drugs) was most common in patients 40–64 years of age (42.2 %) and decreased with increasing age (38.6 and 25.5 % among patients 65–79 and 80+ years of age, respectively). Annual prevalence of levodopa (Fig. 2b) and DA (Fig. 2d) use was similar among men and women.

## Discussion

While unpublished data may regularly be used by the pharmaceutical industry to inform marketing decisions, there are significant benefits to publicly reporting such information. Prescribing patterns serve as markers of practice parameter adherence and response to new scientific evidence. Real-world prescription studies may also identify deviations from standard practice in the form of age, sex, race, or socioeconomic treatment disparities. Our retrospective analyses of inpatients with PD who received pharmacological treatment between January 1, 2001, and December 31, 2012 are, to our knowledge, the first national analysis of trends in PD medication use in the USA. Our primary finding is that there has been a shift in prescribing practices for PD in the USA and that these changes are due in part to emerging safety concerns and evidence of efficacy. Secondary analyses revealed that (1) despite safety concerns, older PD patients were persistently prescribed DAs, (2) use of levodopa and DAs did not greatly differ between men and women over time, and (3) antiparkinson drug polypharmacy was most common in younger PD patients.

DAs gained popularity as the initial pharmacotherapy for PD in the early 2000’s because of concerns over levodopa neurotoxicity and that motor fluctuations in PD were due to levodopa treatment duration [29, 30]. Published in April 2006, the world’s largest professional association of neurologists, the AAN, completed an evidence-based review of the therapies purported to delay the onset of motor fluctuations or decrease motor progression in PD [11]. The expert review reported that levodopa did not accelerate PD progression and that no pharmacological intervention was neuroprotective. Our findings show that USA prescribing trends of antiparkinson drugs, notably DAs, did not immediately change as a result of the AAN’s practice parameter publication. There are several possible reasons for this finding. Continued disagreement regarding the toxicity of levodopa likely contributed to the observed delay in response. The notion that DAs are less toxic (rather than simply less potent) continues to have a strong footing in the scientific literature and lay press [31, 32]. Notwithstanding any disagreement, recent evidence reaffirms the AAN’s finding that levodopa is not toxic [10, 33–35]. Lack of prescriber awareness is another possible reason as to why there was little immediate response to the practice parameter. There is currently no mandate for specialty care for PD, as exists for equally complicated conditions such as cancer. The majority of individuals diagnosed with PD do not have neurologist care, especially in the years immediately following diagnosis [36, 37]. Primary care physician continuing medical education (CME) training may not include specialty practice parameter updates in a timely fashion. Whether lack of awareness of paradigm shifts in PD treatments is associated with worse outcomes will need to be considered in future studies. Finally, despite knowledge of the latest guidelines, sound clinical judgment may have dictated that switching some patients from a DA to levodopa was contraindicated, particularly in patients responding well to a DA or those who had previously experienced intolerable side effects with levodopa.

We next examined inpatient antiparkinson drug use in relation to increasing concerns of adverse events such as cardiovascular complications and impulse control disorders (ICDs). Studies have demonstrated that DAs, specifically ergot derivatives, are associated with the development of cardiac fibrosis and valvular heart disease [3, 6, 8, 9]. Although the majority of DAs prescribed within our study period were nonergot derivatives, it is possible that decreasing use of DAs reflects emerging concerns of cardiac safety with all DAs. Recent reports of increased risk of heart failure with nonergot DAs suggest that researchers are beginning to examine this phenomenon carefully, hopefully providing guidelines that better enable informed use of DAs in patients with cardiovascular disease [4–7]. In addition to cardiovascular concerns, reports of DA-associated ICDs have surged in the last decade [1, 2, 38]. ICDs are characterized by problems in self-control despite personal repercussions and may include pathological gambling, compulsive buying, hypersexuality, binge eating, and punding [1, 2]. While there is currently no black box warning of ICDs with DAs, the precautions section of the pramipexole (Mirapex) label was revised in December 2008 to include information about uncontrollable urges while taking pramipexole, including intense urges to gamble, increased sexual urges, and other intense urges [28]. Increasing knowledge of these risks by clinicians may have contributed in part to the reduced use of DAs after 2007.

Although most physicians avoid DAs in adults >60 years of age over concerns of cognitive impairment [39, 40], a large proportion of individuals >65 years of age in our dataset were prescribed DAs. There was also little change in levodopa or DA use in the most elderly patients. These data may reflect differences in care structure for the oldest PD patients, who are more likely to reside in nursing homes and are least likely to utilize specialty care [41]. Alternatively, older patients or the physicians who care for them may be more averse to the risks of known levodopa-induced side effects (nausea, hallucinations, dyskinesias, and orthostatic hypotension) and instead rely on DAs and other therapies for symptomatic PD. It is also possible that older PD patient populations have a lower susceptibility to ICDs or have more social support to prevent personal and financial consequences of mild dopamine-associated impulsivity, reducing the need to change medications. However, differences in care quality must be considered, as studies have found that older PD patients are undertreated on examination and have undertreatment-related disability [42, 43].

Our study has a number of strengths. Data for this study were derived from a large number of patients spanning multiple treatment centers over 10+ years, with relatively complete pharmacy data. While other studies have investigated antiparkinson drug use cross-sectionally [22, 44], within a single center [16] or within smaller patient populations [20, 22, 44], our study offers broader insight on the influence of practice parameter publication, drug introduction/withdrawal, and increasing knowledge of drug efficacy and safety on prescribing patterns. Since the majority of our study data are derived from urban teaching centers, they are assumed to be highly sensitive to detecting modifications to clinical practice, including changing prescribing patterns in response to new guidelines or evidence.

There are a number of limitations to our study. Due to lacking outpatient drug information, our study was restricted to inpatients prescribed one or more antiparkinson drugs during the study period. This was done to minimize false negatives for antiparkinson treatment. However, because PD is rarely the principal reason for hospital admission [45, 46], we could not account for radical changes in PD regimen that accompanied an admission. Moreover, since differences may exist in the identification and treatment of PD among inpatients and individuals receiving care at academic centers; our findings may not reflect antiparkinson prescribing trends in the general USA PD patient population. Nevertheless, our reported trends provide valuable information on antiparkinson drug use by the oldest and sickest individuals with PD who are hospitalized, many of who (those <65 years of age) are not represented in other large national databases such as Medicare. We could not account for PD disease severity and did not take comorbidities and the use of other medications into account, which may impact prescribing at the individual level. However, our study examined all PD cases over time (rather than a fixed cohort), reducing the potential impact of individual disease progression, comorbidities, and use of other drugs on our results. Unmeasured factors, including drug pricing and pharmaceutical company promotional activity, have certainly influenced antiparkinson drug utilization over time. Therefore, our observed trends in antiparkinson drug use may only be explained in part by examined events. Finally, there are also limitations to our statistical approach. Calculated *p* values from the Wilcoxon-Mann-Whitney test may overstate the significance of changes in drug utilization over time in the presence of clustering in the data, such as might occur if there were a tendency for differences in prescribing practices among hospitals. Percent changes in Table 2, which represent changes in levodopa and DA use for the entire population under study, could mask differential changes by population subgroup; however, trends by age, sex, race, and census region shown in Supplementary Table 1 did not generally show marked differences among these subgroups.

Despite study limitations, we demonstrate that changes in the use of antiparkinson drugs in the USA have occurred over time and that these changes may reflect increasing knowledge of drug safety and efficacy. Future studies that examine the impact of care structure and quality on prescribing practices, particularly with regard to barriers to the dissemination, acceptance, and adoption clinical guidelines, as well as studies which investigate outcomes associated with drug choice in select PD populations are needed.

## Electronic supplementary material

ESM 1 (DOCX 586 kb).
