# Supplementary material for: Trends in inpatient antiparkinson drug use in the USA, 2001–2012
Source: Eur J Clin Pharmacol. 2015 Jun 18;71(8):1011–9. doi: 10.1007/s00228-015-1881-4 (PMC4500853; doi:10.1007/s00228-015-1881-4)
Supplement: Supplementary file 1 — (DOCX 586 kb). [file 228_2015_1881_MOESM1_ESM.docx]

**Trends in inpatient antiparkinson drug use in the United States, 2001-2012**

European Journal of Clinical Pharmacology

James A.G. Crispo, MSc, Yannick Fortin, MA, Dylan P. Thibault, MS, Matthew Emons, MD, MBA, Lise M. Bjerre, MD, PhD, MCFP, Dafna E. Kohen, PhD, Santiago Perez Lloret, MD, PhD, CPI, Donald Mattison, MD, MS, Allison W. Willis, MD, MS, and Daniel Krewski, PhD, MHA.

**Supplementary Table 1.** Study cohort demographics, care setting census regions, and prevalence of antiparkinson drug use by study year.


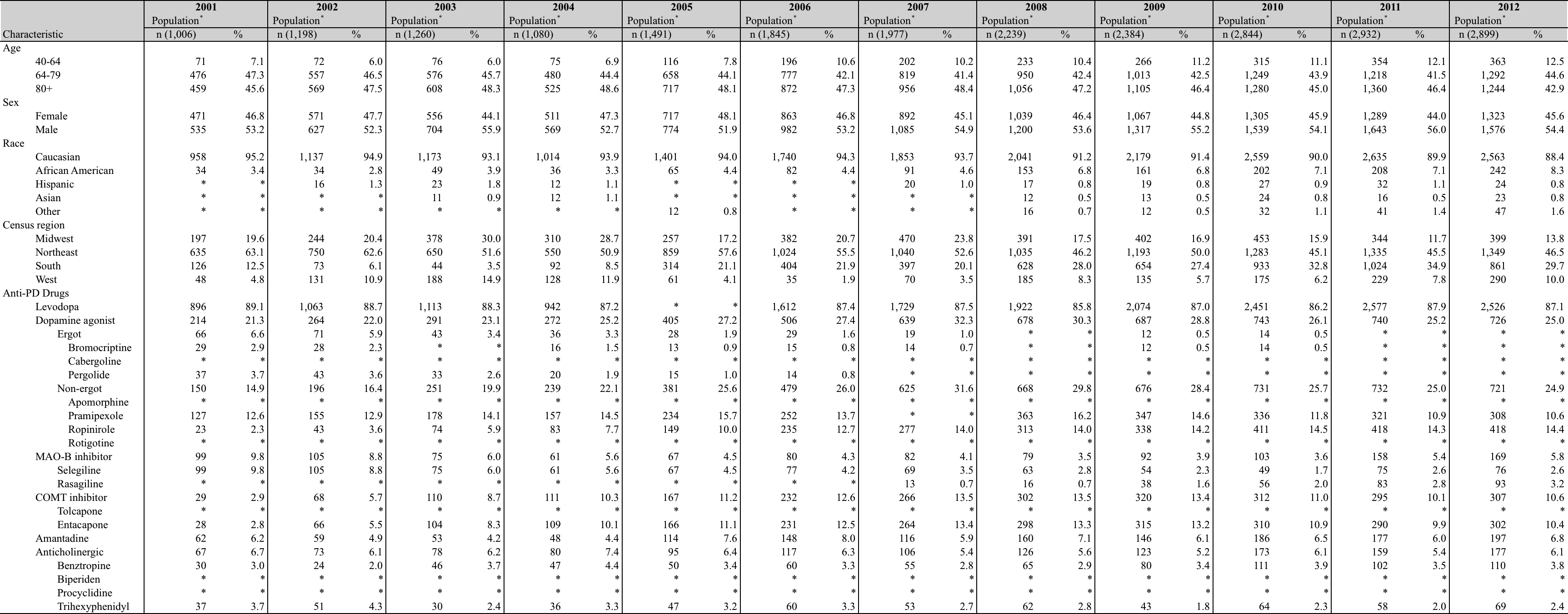


Abbreviations: COMT, catechol-o-methyltransferase; MAO-B, monoamine oxidase-B; PD, Parkinson disease.

*To reduce the risk of individual identification of persons, results for cells where tabulated data is less than or equal to 10 is not shown.

**Corresponding Author:**

James Crispo MSc, Canadian Fulbright Student, University of Pennsylvania; PhD Candidate, Population Health, University of Ottawa

Room 118, 850 Peter Morand Crescent, Ottawa, Ontario, Canada K1G 3Z7; Tel: (613) 850-7585; Email: jcris021@uottawa.ca

**Supplementary Table 2.** Reasons for hospitalization of individuals with Parkinson disease, 2001-2012

|  |  | |
| --- | --- | --- |
|  | Frequency^*^ |  |
| Principal Diagnosis (by ICD-9 category) | n (54,345) | % |
| Infectious and parasitic diseases (001-139) | 2,505 | 4.6 |
| Neoplasms (140-239) | 804 | 1.5 |
| Endocrine, nutritional and metabolic diseases, and immunity disorders (240-279) | 1,967 | 3.6 |
| Diseases of the blood and blood-forming organs (280-289) | 469 | 0.9 |
| Mental disorders (290-319) | 1,958 | 3.6 |
| Diseases of the nervous system (320-359) | 3,745 | 6.9 |
| Diseases of the sense organs (360-389) | 107 | 0.2 |
| Diseases of the circulatory system (390-459) | 8,359 | 15.4 |
| Diseases of the respiratory system (460-519) | 6,201 | 11.4 |
| Diseases of the digestive system (520-579) | 3,229 | 5.9 |
| Diseases of the genitourinary system (580-629) | 3,639 | 6.7 |
| Complications of pregnancy, childbirth, and the puerperium (630-679) | * | * |
| Diseases of the skin and subcutaneous tissue (680-709) | 943 | 1.7 |
| Diseases of the musculoskeletal system and connective tissue (710-739) | 2,438 | 4.5 |
| Congenital anomalies (740-759) | * | * |
| Certain conditions originating in the perinatal period (760-779) | * | * |
| Symptoms, signs, and ill-defined conditions (780-799) | 6,752 | 12.4 |
| Injury and poisoning (800-999) | 4,526 | 8.3 |
| E Codes (all codes) | * | * |
| V Codes (all codes) | 1,989 | 3.7 |
| Missing | 4,588 | 8.4 |

Abbreviation: ICD-9, International Classification of Diseases, Ninth Revision.

*To reduce the risk of individual identification of persons, results for cells where tabulated data is less than or equal to 100 is not shown. Individuals may have more than one recorded principal diagnosis per inpatient encounter.
